# Reducing Polypharmacy-Related Adverse Outcomes in Older Adults with Chronic Kidney Disease: A Retrospective Cohort Study of a Digitally Mediated Pharmacist Intervention

**DOI:** 10.3390/jcm15031128

**Published:** 2026-02-01

**Authors:** Keren Dopelt, Ori Mayer, Adir Dagan, Guy Melamed, Aviva Ben-Baruch, Inbal Yifrach-Damari, Tamar Ritte

**Affiliations:** 1 Department of Public Health, Ashkelon Academic College, Ashkelon 78211, Israel; dopelt@bgu.ac.il; 2 Department of Cell and Developmental Biology, Gray Faculty of Medicine, Tel-Aviv University, Tel Aviv 69978, Israel; 3 Kahn-Sagol-Maccabi (KSM) Research & Innovation Institute, Maccabi Healthcare Services, Tel Aviv 67062, Israel; 4 Gray Faculty of Health Sciences, Tel-Aviv University, Tel Aviv 69978, Israel; 5 Department of Quality Management, Health Division, Maccabi Healthcare Services, Tel Aviv 67062, Israel; 6 Department of Pharmacy and Clinical Pharmacology, Health Division, Maccabi Healthcare Services, Tel Aviv 67062, Israel

**Keywords:** chronic kidney disease, polypharmacy, pharmacist-led intervention, medication safety, adverse outcomes, aged

## Abstract

**Background/Objectives**: Older adults with chronic kidney disease (CKD) are particularly vulnerable to polypharmacy-related adverse outcomes due to altered pharmacokinetics, multimorbidity, and increased susceptibility to medication-related harm. Polypharmacy in CKD is associated with falls, hospitalizations, and functional decline. Clinical pharmacist-led medication reviews may mitigate these risks; however, access barriers limit their implementation in routine care. To evaluate the clinical impact of a digitally mediated pharmacist consultation service on medication burden, fall risk, healthcare utilization, and resource use among older adults with CKD and polypharmacy. **Methods**: We conducted a retrospective cohort study using anonymized electronic medical records from a large integrated healthcare organization. Adults aged ≥ 65 years with CKD and polypharmacy (≥8 chronic medications) were included. Patients receiving a structured digital medication review by a clinical pharmacist, delivered via the primary care physician, were compared with a comparable control group of eligible patients who did not receive the intervention during the study period. Outcomes included changes in medication use, fall risk, renal function, and healthcare utilization. **Results**: Among 6124 eligible patients (1226 intervention; 4898 control), pharmacist consultation was associated with a modest but clinically meaningful reduction in medication burden and a higher likelihood of fall-risk reduction compared with controls. Decreases in outpatient healthcare utilization were also observed following the intervention. Renal function decline was similar between groups. **Conclusions**: A digitally mediated, physician-integrated pharmacist consultation may reduce polypharmacy-related risks and adverse outcomes in older adults with CKD. This model offers a scalable approach to improving medication safety in a high-risk CKD population while minimizing reliance on patient digital engagement.

## 1. Introduction

Chronic kidney disease (CKD) represents a growing public health challenge worldwide, particularly among older adults, and is associated with substantial clinical complexity, high healthcare utilization, and adverse health outcomes [1,2]. The burden of CKD is amplified by population aging, multimorbidity, and polypharmacy [3,4,5].

Population aging is accompanied by rising rates of chronic disease, falls, and polypharmacy, leading to increased healthcare expenditures [6,7,8]. Among individuals with CKD, these challenges are particularly pronounced due to altered pharmacokinetics, reduced renal clearance, and heightened susceptibility to medication-related harm [9,10].

Polypharmacy is defined as the concurrent use of multiple medications, often five or more, to treat one or multiple coexisting conditions [11]. Its management poses a significant challenge for healthcare providers [12] and has been consistently associated with poor clinical outcomes and an increased risk of drug-related problems [13,14]. In patients with CKD, polypharmacy is further linked to inappropriate dosing, exposure to nephrotoxic agents, accelerated renal decline, and increased risks of hospitalization and mortality [15,16].

Beyond medication-related harm, polypharmacy has far-reaching clinical consequences for older adults, particularly those with CKD, including recurrent falls, hospitalizations, adverse drug effects, deterioration of kidney function, and impaired quality of life [2,9]. Falls and functional decline are especially concerning adverse outcomes in older adults with CKD, given their strong association with disability, loss of independence, and increased healthcare utilization [17].

In recent years, the use of digital health services has expanded substantially, with telepharmacy emerging as an important component of modern healthcare delivery. Evidence suggests that tele-pharmacy can improve clinical outcomes, reduce medication errors, and lower healthcare costs [18,19,20]. However, many older adults face barriers to accessing or independently using digital health platforms, particularly those with chronic illness and functional limitations. In CKD populations, limited digital literacy and high clinical complexity may further constrain the effectiveness of patient-facing digital interventions [21,22].

To address the need for medication management while minimizing reliance on patient digital literacy, a consulting service was developed for elderly individuals in healthcare settings in Israel. The service targets older adults with CKD and polypharmacy, a population at particularly high risk for medication-related adverse outcomes. Using routinely generated analytics reports, eligible patients are identified and referred to a clinical pharmacist, who conducts a structured medication review and provides individualized recommendations within the electronic medical record. These recommendations are communicated digitally to the primary care physician, who evaluates and implements treatment adjustments in collaboration with the patient.

Although tele-pharmacy consultations have demonstrated potential benefits, evidence remains limited regarding models in which pharmacist input is mediated through the primary care physician rather than delivered directly to patients. Moreover, data are scarce on the clinical impact of such models on CKD-relevant adverse outcomes, including medication burden, fall risk, renal function, and healthcare utilization. Therefore, this retrospective cohort study aimed to evaluate whether a digitally mediated, physician-integrated pharmacist consultation is associated with reduced polypharmacy and improved clinical outcomes, including fall risk and healthcare utilization, among older adults with CKD.

## 2. Materials and Methods

### 2.1. Setting and Intervention Description

Maccabi Health Services is the second-largest health organization in Israel. As of 2023, approximately 15,000 patients aged 65 years and older with CKD were listed in Maccabi’s polypharmacy registry. For this patient population, an online clinical pharmacist consultation is performed biannually.

Within Maccabi Health Services, approximately 40 clinical pharmacists (corresponding to fewer than 30 full-time equivalent positions) are employed across all geographic districts. Clinical pharmacists are responsible for promoting rational medication use, providing ongoing pharmacotherapy consultation to healthcare professionals, participating in multidisciplinary teams, and conducting medication reviews in cases of polypharmacy.

The intervention targets older adults (≥65 years) who are prescribed eight or more chronic medications and have an estimated glomerular filtration rate (eGFR) of 60 mL/min or less or microalbuminuria in their two most recent laboratory results. The intervention involves a comprehensive and structured medication review conducted by a clinical pharmacist. The medication therapy work-up is guided by a pharmaceutical care approach, consistent with the framework described by Cipolle, Strand, and Morley [23], and is applied in a standardized manner across clinical pharmacists. The primary aim is to systematically evaluate each patient’s chronic medication regimen and reduce it to the minimal number of essential and clinically justified medications, taking into account comorbidities and individual clinical needs.

The pharmacist’s workup includes a systematic review of the complete active medication list, relevant laboratory values (including renal function), documented diagnoses, and recent clinical events. Clinical pharmacists have full access to the patient’s comprehensive electronic medical record, which integrates data from all treating physicians within the organization, as well as hospital discharge summaries, outpatient clinic documentation, laboratory results, and medication dispensing records. Particular attention is given to medications associated with increased fall risk, nephrotoxicity, and age-related adverse effects.

This review systematically addresses multiple domains, including the necessity of therapy; safety (with consideration of dose adjustments based on renal and hepatic function, approved indications, patient age, and potential adverse drug reactions); drug–drug and drug–diagnosis interactions; monitoring requirements; therapeutic effectiveness; convenience; and patient adherence.

The assessment is guided by evidence-based resources, including Micromedex, UpToDate, MedicineComplete, the Israeli Ministry of Health’s Drug Database, and the U.S. Food and Drug Administration (FDA). Additional tools, including clinical calculators (e.g., FRAX, SCORE) and current official clinical guidelines (e.g., Beers Criteria), are utilized to support informed decision-making. The digital pharmacist consultation note follows a structured format within the electronic health record and includes predefined sections for identified medication-related problems, clinical rationale, and specific, actionable recommendations (e.g., medication discontinuation, dose adjustment, substitution, or monitoring). Upon completion of the review, pharmacist recommendations are communicated to the treating physician via an automated alert in the electronic medical record, indicating the availability of a clinical pharmacist consultation. The physician subsequently discusses and implements treatment decisions with the patient.

### 2.2. Participants

The study included 6124 participants, with 1226 in the intervention group and 4898 in the control group. The intervention group consisted of patients who received a digitally mediated clinical pharmacist consultation, whereas the control group comprised eligible patients who did not receive the intervention during the study period.

The control group consisted of patients who met the same eligibility criteria as the intervention group but did not receive a pharmacist consultation during the study period. No formal matching procedure was applied; baseline demographic and clinical characteristics were compared to assess group comparability.

Eligibility criteria included age ≥ 65 years, current use of eight or more chronic medications, and an eGFR value of 60 mL/min or less or microalbuminuria in the two most recent measurements. Patients with incomplete or inconsistent demographic data, duplicate entries, or logical discrepancies (e.g., conflicting records on the same date) were excluded from the analysis.

### 2.3. Statistical Methods

This retrospective cohort study was conducted using anonymized electronic medical records obtained from Maccabi Health Services. The study period is 2021–2023. The study period was selected to reflect routine clinical implementation of the digitally mediated pharmacist consultation service following its system-wide adoption and stabilization. Rigorous data validation procedures were applied, including detection of duplicate patient records, logical consistency checks, and identification of outliers according to predefined rules. Missing data were retained to minimize selection bias. The imbalance between the intervention and control groups reflects real-world service utilization: only a subset of eligible patients received the pharmacist consultation during the study period, while the remaining eligible patients comprised the control group. Analyses were conducted using the full eligible population to maximize statistical power. Between-group comparisons were performed without weighting or matching, and baseline characteristics were examined to assess comparability between groups. Chronic kidney disease severity was assessed using estimated glomerular filtration rate (eGFR) values derived from laboratory data. Fall risk was evaluated using routinely documented clinical indicators within the electronic health record, including prior documented falls, use of fall-risk-associated medications, and recorded fall-risk status. Actual fall events were not systematically captured for all patients and were therefore not analyzed as a separate outcome. An osteoporosis indicator was defined based on documented diagnoses and medication proxies available in the electronic medical record; bone mineral density T-scores were not routinely available to the pharmacist at the time of consultation.

All statistical analyses were performed using R software version 4.5.0 (Vienna, Austria) with the base, dplyr, and gtsummary packages. Continuous variables were summarized using both mean ± standard deviation (SD) and median (Q1, Q3) values. Categorical and binary variables were presented as frequencies and percentages (n, %). Between-group comparisons were performed using the Wilcoxon rank-sum test for continuous and ordinal variables, the proportion test for binary variables, and the chi-square test for categorical variables. Within-group (paired) analyses were conducted separately for the intervention and control groups to assess changes in pre- and post-counseling outcomes. The paired Wilcoxon test was applied to continuous variables, with paired *t*-tests used to estimate within-subject differences where appropriate.

### 2.4. Ethics

All the data were retrieved from the medical records without identifying details. The current study was approved by the Maccabi Health Services ethical committee (approval number MHS-0081-23, dated 9 January 2024), with an exemption from informed consent requirements, as it involved retrospective analysis of anonymized data with no direct patient contact. Data were obtained after receiving the Institutional Ethical Committee approval.

## 3. Results

### 3.1. Participants and Baseline Characteristics

Of the 6664 eligible patients, 6124 met the inclusion criteria and were included in the analysis. Among them, 1226 patients received a digital pharmacist consultation (intervention group), and 4898 eligible patients who did not receive the consultation served as the control group. Baseline characteristics were largely comparable between groups. Patients’ demographic and medical characteristics are summarized in Table 1. The mean age was 80 years (standard deviation [SD] 7), and 52% were female. Minor but statistically significant differences were observed in socioeconomic status and area of residence, although these were not considered clinically meaningful. Participants residing in peripheral areas were overrepresented in the intervention group, possibly reflecting greater use of remote consultation services in these regions, which often face increased geographic, transportation, and availability barriers to accessing in-person care. The median follow-up times were 21 months (interquartile range [IQR], 11–29 months) prior to the consultation and 16 months (IQR, 7–25 months) after the consultation. All patients met the criteria for polypharmacy, with a median number of regularly taken medications of 11 (IQR 9–13) in the control group and 12 (IQR 10–14) in the intervention group (*p* < 0.001). All patients met the criteria for CKD. Chronic kidney disease status was defined based on physician-documented CKD diagnosis and/or laboratory criteria (eGFR ≤ 60 mL/min/1.73 m^2^ or microalbuminuria in the two most recent measurements), rather than on small between-group differences in baseline eGFR values. The mean estimated glomerular filtration rate (eGFR) was 58 ± 19 mL/min/1.73 m^2^ in the intervention group and 64 ± 17 mL/min/1.73 m^2^ in the control group (*p* < 0.001). Although statistically significant, this difference was considered clinically modest. The distribution of severe disease registry classifications was similar between groups, whereas baseline fall-risk registry scores showed statistically significant but minor differences. These differences were small in magnitude and unlikely to be clinically meaningful.

### 3.2. Medication Use Outcomes

Patients in the intervention group initiated a median of 5 (IQR 0–10) new chronic medications following the consultation, compared with 3 (IQR 0–7, *p* < 0.001; Table 2) in the control group. The median number of discontinued medications was higher in the intervention group than in the control group (1 [IQR 0–3] vs. 0 [IQR 0–2], *p* < 0.001). Within the intervention group, the median number of newly initiated medications decreased from 7 (IQR 4–13) before the consultation to 5 (IQR 0–10) after (*p* < 0.001; Table 3), while the median number of discontinued medications decreased from 2 (IQR 0–6) to 1 (IQR 0–3) (*p* < 0.001). In the control group, a smaller reduction in newly initiated medications was observed (from 4 [IQR 2–8] to 3 [IQR 0–7], *p* < 0.001), with minimal change in discontinued medications (1 [IQR 0–3] before vs. 0 [IQR 0–2] after, *p* < 0.001). Despite higher rates of medication initiation, increased discontinuation resulted in a net reduction in overall medication burden in the intervention group. Overall, these changes resulted in a net reduction in one medication in the median total number of chronic prescriptions in the intervention group compared with the control group (*p* < 0.001; Table 2). Medication reduction reflects a process of regimen optimization, including discontinuation of potentially inappropriate or high-risk medications and dose adjustments based on age and renal function, rather than simply reducing the medication count.

Within-group analyses demonstrated significant pre–post changes in medication initiation and discontinuation in both groups, with more pronounced effects observed in the intervention group (Table 3). Medication initiation and discontinuation counts reflect cumulative changes that occur over the entire follow-up period, both before and after the pharmacist consultation (median follow-up: 21 months pre-intervention and 16 months post-intervention).

### 3.3. Clinical Outcomes

While no between-group difference was observed in the prevalence of osteopenia or major fractures following the intervention, a small but statistically significant absolute reduction of 0.3 percentage points in major fractures was noted within the intervention group (Table 4, *p* = 0.044), whereas no such change occurred in the control group (*p* = 0.40). Both groups demonstrated a similar significant decline of 2 mL/min/1.73 m^2^ in estimated glomerular filtration rate over the study period (*p* < 0.001). No clinically meaningful between-group difference in renal function trajectory was observed.

Additionally, changes were observed in the distribution of fall risk categories before and after the intervention in both groups. When analyzed at the individual level, a higher proportion of patients in the intervention group experienced a decrease in fall risk compared with the control group (Table 5, 6.5% vs. 4.8%, *p* = 0.016). Fall risk categories were derived from routinely documented clinical indicators within the electronic health record; confirmed fall events were not systematically captured for all patients. No significant differences were observed in the proportions of patients with increased or stable fall risk.

### 3.4. Healthcare Utilization and Costs

During the study period, emergency department visits were rare and therefore not analyzed separately. Between-group comparisons revealed a higher number of primary care visits in the intervention group compared to the control group, with medians of 14 (IQR, 7–22) and 12 (IQR, 6–18), respectively (Table 6, *p* < 0.001). A modest but statistically significant increase was also observed in secondary care visits, 4 (IQR 1–9) versus 4 (IQR 1–8, *p* < 0.001), and in visits to other health professionals, 5 (IQR 2–11) versus 3 (IQR 1–8, *p* < 0.001). Hospitalization outcomes were analyzed descriptively due to low event rates. Healthcare cost analyses were based on recorded healthcare utilization data; the cost of the pharmacist consultation was not included, as this service is embedded within standard organizational care pathways and is not billed separately. Within-group analyses revealed decreases across most categories of healthcare utilization following the intervention.

In the intervention group, median primary care visits declined from 20 (IQR 14–29) before the consultation to 14 (IQR 7–22) after (Table 7, *p* < 0.001); secondary care visits decreased from 7 (IQR 3–12) to 4 (IQR 1–9, *p* < 0.001); and visits to other health professionals declined from 8 (IQR 4–15) to 5 (IQR 2–11, *p* < 0.001). Similar trends were observed in the control group, where primary care visits fell from 15 (IQR 10–21) to 12 (IQR 6–18, *p* < 0.001), secondary care visits from 6 (IQR 3–10) to 4 (IQR 1–8, *p* < 0.001), and visits to other health professionals from 5 (IQR 3–10) to 3 (IQR 1–8, *p* < 0.001).

## 4. Discussion

This study evaluated the impact of a digitally mediated clinical pharmacist consultation service on medication management among older adults with CKD and polypharmacy. Consistent with previous research, our findings demonstrate modest but clinically meaningful reductions in overall medication burden and fall risk, alongside a decrease in selected outpatient healthcare utilization following the intervention. Polypharmacy is particularly problematic in CKD due to altered pharmacokinetics, increased susceptibility to adverse drug events, and complex multimorbidity [1,10]. Prior studies have shown that reducing inappropriate medications can improve safety and slow functional and renal decline [15,24], supporting the direction and clinical relevance of the observed benefits in our cohort.

In this study, the digitally mediated pharmacist consultation was associated with a modest but clinically meaningful net reduction in medication burden among older adults with CKD. Although patients in the intervention group initiated more medications than controls, this was offset by higher rates of medication discontinuation, resulting in an overall reduction in chronic prescriptions. This pattern is consistent with prior pharmacist-led medication review studies, which emphasize medication optimization and deprescribing rather than simple medication reduction [25]. However, the magnitude of medication reduction observed in our study was smaller than that reported in some controlled deprescribing trials [26], likely reflecting the high clinical complexity, multimorbidity, and real-world setting of the CKD population examined [27,28]. This interpretation is further supported by realist syntheses showing that multidisciplinary medication review and deprescribing interventions in primary care tend to yield modest but sustainable effects when embedded in routine clinical workflows and focused on individualized optimization rather than maximal medication reduction [29]. Our findings are also consistent with structured deprescribing frameworks, such as the STOPP/START criteria, which emphasize medication optimization based on clinical appropriateness and patient characteristics rather than solely on reduction in medication count [30].

Research showed that reducing the use of potentially inappropriate medications improves treatment quality among older adults [31]. Recent meta-analyses, specifically in CKD populations, indicate that pharmacist involvement improves dosing appropriateness, reduces nephrotoxic exposure, and decreases the risk of hospitalization [16,32,33]. In addition, emerging evidence suggests that pharmacist-led digital or mobile health interventions for CKD patients can improve medication adherence and surrogate clinical indicators such as eGFR, demonstrating the feasibility of pharmacist support delivered through remote platforms [34]. The present findings extend this evidence by demonstrating that such reviews can be effectively delivered through a digitally mediated, physician-integrated model.

A key finding of the present study was a higher proportion of patients experiencing a reduction in fall risk following the intervention than in controls. Although the absolute difference was modest, this finding aligns with meta-analyses indicating that medication review and deprescribing interventions can reduce fall risk among older adults [17]. Notably, most prior studies evaluated face-to-face or patient-directed interventions, whereas our results demonstrate that a physician-mediated, digitally delivered pharmacist consultation can yield benefits comparable to those of face-to-face or patient-directed interventions [18]. The relatively small effect observed may reflect conservative implementation of recommendations by physicians and the high baseline frailty of older adults with CKD, consistent with prior work showing that deprescribing in frail populations often yields attenuated effects [35,36,37,38].

Mediation through a primary care physician is particularly relevant in the context of digital health equity. Literature suggests that telehealth interventions can inadvertently widen disparities if they require high digital literacy [21,22]. By contrast, models that integrate tele-pharmacy into existing provider workflows, rather than placing responsibility on the patient, are associated with higher uptake and improved continuity of care [18,39,40]. Our results align with these findings and suggest that a physician-mediated digital consultation may promote equitable access to pharmacist expertise. Moreover, this model may also benefit from patients’ trust in their primary care physician, as higher levels of trust in the healthcare system have been shown to be associated with greater acceptance of recommended health interventions [41].

The reduction in outpatient healthcare utilization observed after the intervention, even when accounting for baseline differences, is noteworthy given the typically high resource utilization in this population. Although post-intervention healthcare utilization remained higher in the intervention group compared with the control group, this likely reflects greater baseline clinical complexity among patients selected for pharmacist consultation, rather than increased healthcare dependence attributable to the intervention itself. In contrast to some interventional studies reporting stabilization or improvement in renal function following pharmacist-led or digital medication management interventions [34], we observed a similar decline in eGFR in both groups over time. This finding is not unexpected given the advanced age of the study population and the natural progression of chronic kidney disease, in which renal function often continues to decline despite optimized care [42,43]. These results suggest that the primary benefit of the intervention lies in improved medication safety and risk reduction rather than disease modification, consistent with prior work emphasizing deprescribing and medication optimization as safety-focused strategies in older adults with CKD [15]. Although the absolute effects were modest, recent deprescribing trials emphasize that incremental reductions in medication burden can lead to meaningful improvements in physical function, fall risk, and symptom control [17].

### 4.1. Importance to Professionals

A digitally mediated pharmacist consultation service expands access to clinical medication review for patients who might not otherwise seek or be able to obtain pharmacological guidance independently. Evidence suggests that many older adults are reluctant or unable to engage with digital health platforms, which may lead to reduced use of essential healthcare services [44]. By routing consultations through primary care physicians, this service leverages the physician’s role as a clinical and organizational leader [45] while simultaneously minimizing reliance on patient digital literacy, thereby ensuring more equitable access to pharmacist expertise. This model is designed to support the health of older adults with CKD by promoting safer medication management, with the potential to improve quality of life and reduce health disparities arising from digital gaps in healthcare delivery.

### 4.2. Study Limitations

This study has several limitations. First, its retrospective and observational design limits the ability to infer causal relationships between the pharmacist consultation and clinical outcomes, as unmeasured confounding factors such as differences in baseline health status, physician practice patterns, or patient engagement may have influenced the results. Despite rigorous data validation, residual confounding cannot be excluded, and some potentially important variables, such as frailty, cognitive function, and social support, were not available in the dataset. Furthermore, several outcomes, including emergency department visits and hospitalizations, were infrequent and therefore underpowered for statistical analysis, while patient-centered measures such as medication adherence, satisfaction, and quality of life were not assessed. The reliance on routinely collected electronic medical record data also raises the possibility of misclassification or incomplete recording of medications and diagnoses. Additionally, the follow-up period may have been insufficient to capture the long-term effects on renal function progression or morbidity. Moreover, some statistically significant differences between groups may be partly attributable to the large sample size rather than reflecting clinically meaningful effects; therefore, interpretation should consider both effect sizes and clinical relevance. Finally, as the study was conducted within a single healthcare organization in Israel, the findings may not be generalizable to other healthcare systems with different digital infrastructures, pharmacist roles, or prescribing practices.

## 5. Conclusions

This study suggests that a structured digital pharmacist consultation service may improve medication management among older adults with CKD and polypharmacy. The intervention was associated with modest but meaningful reductions in medication load, fall risk, and selected outpatient healthcare utilization, underscoring the value of integrating clinical pharmacists into digital care pathways to enhance medication safety and optimize therapy for older adults. Future research should confirm these findings in prospective or randomized studies and assess long-term outcomes, such as renal function, hospitalization rates, and mortality. Studies that include patient-reported outcomes, such as quality of life and medication adherence, would further clarify the broader impact of this intervention and guide its implementation in other healthcare settings.

## Figures and Tables

**Table 1:** Sample Characteristics.

Characteristic	Statistic	Control	Study	Overall	*p*-Value
		N = 4898	N = 1226	N = 6124	
Demographic Characteristics					
Age	Mean (SD)	80 (7)	80 (7)	80 (7)	>0.9
Sex					>0.9
Female	n (%)	2560 (52%)	640 (52%)	3200 (52%)	
Male	n (%)	2338 (48%)	586 (48%)	2924 (48%)	
Socioeconomic Status					<0.001
low	n (%)	964 (20%)	346 (28%)	1310 (22%)	
High	n (%)	3905 (80%)	869 (72%)	4774 (78%)	
Unknown	n	29	11	40	
District					<0.001
North	n (%)	669 (14%)	219 (18%)	888 (15%)	
Jerusalem and Shfela	n (%)	1139 (23%)	223 (18%)	1362 (22%)	
Hasharon	n (%)	1164 (24%)	280 (23%)	1444 (24%)	
Central	n (%)	1596 (33%)	353 (29%)	1949 (32%)	
South	n (%)	330 (6.7%)	151 (12%)	481 (7.9%)	
Follow-up Years Before Consultation	Median (Q1–Q3)	1.73 (0.93–2.45)	1.73 (0.93–2.45)	1.73 (0.93–2.45)	>0.9
Follow-up Years After Consultation	Median (Q1–Q3)	1.27 (0.55–2.06)	1.27 (0.55–2.06)	1.27 (0.55–2.06)	>0.9
Medical Characteristics					
Severe Disease Indicator					0.5
No Disease	n (%)	3909 (80%)	971 (79%)	4880 (80%)	
Inactive Disease	n (%)	696 (14%)	168 (14%)	864 (14%)	
Active Disease	n (%)	293 (6.0%)	87 (7.1%)	380 (6.2%)	
Osteoporosis Indicator	n (%)	2254 (46%)	621 (51%)	2875 (47%)	0.004
Chronic Prescriptions	Median (Q1–Q3)	11.00 (9.00–13.00)	12.00 (10.00–14.00)	11.00 (9.00–13.00)	<0.001
eGFR Before	Mean (SD)	64 (17)	58 (19)	63 (17)	<0.001
Unknown	n	1136	133	1269	
Fall Risk Before					0.003
No Risk	n (%)	2399 (49%)	568 (46%)	2967 (48%)	
Low	n (%)	1010 (21%)	217 (18%)	1227 (20%)	
Medium	n (%)	826 (17%)	230 (19%)	1056 (17%)	
High	n (%)	663 (14%)	211 (17%)	874 (14%)	

**Table 2:** Between Treatment Groups: Medication Use Outcomes.

Characteristic	Statistic	Control	Study	Overall	*p*-Value
		N = 4898	N = 1226	N = 6124	
Number of Chronic Prescriptions Before Consultation	Median (Q1–Q3)	3 (1–5)	4 (2–8)	3 (1–6)	<0.001
Number of Chronic Prescriptions After Consultation	Median (Q1–Q3)	2 (0–5)	3 (0–6)	2 (0–5)	<0.001
Diff Number of Chronic Prescriptions	Median (Q1–Q3)	0 (−2–1)	−1 (−3–1)	0 (−2–1)	<0.001
Number of Chronic Prescriptions Start After Consultation	Median (Q1–Q3)	3 (0–7)	5 (0–10)	3 (0–7)	<0.001
Number of Chronic Prescriptions Stopped After Consultation	Median (Q1–Q3)	0 (0–2)	1 (0–3)	0 (0–2)	<0.001
Diff Chronic Prescriptions Start	Median (Q1–Q3)	−1 (−5–2)	−2 (−8–2)	−1 (−5–2)	<0.001
Unknown	n	171	16	187	
Chronic Prescriptions Change Category					<0.001
−	n (%)	2483 (53%)	729 (60%)	3212 (54%)	
0	n (%)	449 (9.5%)	89 (7.4%)	538 (9.1%)	
+	n (%)	1795 (38%)	392 (32%)	2187 (37%)	
Unknown	n	171	16	187	

**Table 3:** Within-Group Pre–Post Changes in Initiated and Discontinued Chronic Medications Over the Pre- and Post-Intervention Follow-Up Periods.

**Study**				
**Characteristic**	**Statistic**	**Before**	**After**	***p*** **-Value**
		**N = 1226**	**N = 1226**	
Number of Chronic Prescriptions Start	Median (Q1–Q3)	7 (4–13)	5 (0–10)	<0.001
Number of Chronic Prescriptions Stopped	Median (Q1–Q3)	2 (0–6)	1 (0–3)	<0.001
**Control**				
**Characteristic**	**Statistic**	**Before**	**After**	***p*** **-Value**
		**N = 4898**	**N = 4898**	
Number of Start Permanent	Median (Q1–Q3)	4 (2–8)	3 (0–7)	<0.001
Number of Stop Permanent	Median (Q1–Q3)	1 (0–3)	0 (0–2)	<0.001

**Table 4:** Between Treatment Group Clinical Outcomes.

Characteristic	Statistic	Control	Study	Overall	*p*-Value
		N = 4898	N = 1226	N = 6124	
Is Fracture Osteopenia after	n (%)	5 (0.1%)	0 (0%)	5 (<0.1%)	0.6
Is Fracture Major after	n (%)	70 (1.4%)	15 (1.2%)	85 (1.4%)	0.7
eGFR after	Mean (SD)	62 (17)	56 (19)	61 (17)	<0.001
Unknown	n	1500	295	1795	
Fall Risk Increase	n (%)	641 (13%)	166 (14%)	807 (13%)	0.7
Fall Risk No Change	n (%)	4023 (82%)	980 (80%)	5003 (82%)	0.082
Fall Risk Decrease	n (%)	234 (4.8%)	80 (6.5%)	314 (5.1%)	0.016
Fall Risk after					0.004
No Risk	n (%)	1996 (41%)	471 (38%)	2467 (40%)	
Low	n (%)	1244 (25%)	265 (22%)	1509 (25%)	
Medium	n (%)	890 (18%)	267 (22%)	1157 (19%)	
High	n (%)	768 (16%)	223 (18%)	991 (16%)	

**Table 5:** Within Treatment Groups Clinical Outcomes.

**Within-Subject Comparison (Study)**				
**Characteristic**	**Statistic**	**Before**	**After**	***p*** **-Value**
		**N = 1226**	**N = 1226**	
Fracture Occurrence—Osteopenia-related	n (%)	2 (0.2%)	0 (0%)	0.1
Fracture Occurrence Major	n (%)	28 (2.3%)	15 (1.2%)	0.044
eGFR				<0.001
Mean (SD)		58 (19)	56 (19)	
Unknown		133	295	
Fall Risk				<0.001
No Risk	n (%)	568 (46%)	471 (38%)	
Low	n (%)	217 (18%)	265 (22%)	
Medium	n (%)	230 (19%)	267 (22%)	
High	n (%)	211 (17%)	223 (18%)	
**Within-Subject Comparison (Control)**				
**Characteristic**	**Statistic**	**Before**	**After**	***p*** **-Value**
		**N = 4898**	**N = 4898**	
Fracture Occurrence—Osteopenia-related	n (%)	7 (0.1%)	5 (0.1%)	0.6
Fracture Occurrence Major	n (%)	81 (1.7%)	70 (1.4%)	0.4
eGFR	Mean (SD)	64 (17)	62 (17)	<0.001
Unknown	n	1136	1500	
Fall Risk				<0.001
No Risk	n (%)	2399 (49%)	1996 (41%)	
Low	n (%)	1010 (21%)	1244 (25%)	
Medium	n (%)	826 (17%)	890 (18%)	
High	n (%)	663 (14%)	768 (16%)	

**Table 6:** Between Treatment Groups: Healthcare Utilization and Costs.

Characteristic	Statistic	Control	Study	Overall	*p*-Value
		N = 4898	N = 1226	N = 6124	
Ed Visits after	Median (Q1–Q3)	0 (0–0)	0 (0–0)	0 (0–0)	0.003
Health Professions after	Median (Q1–Q3)	3 (1–8)	5 (2–11)	4 (1–9)	<0.001
Secondary Care after	Median (Q1–Q3)	4 (1–8)	4 (1–9)	4 (1–8)	<0.001
Primary Care after	Median (Q1–Q3)	12 (6–18)	14 (7–22)	12 (6–19)	<0.001
Sum Hospitalization Days after	Median (Q1–Q3)	6 (3–13)	5 (3–14)	6 (3–13)	0.8
Unknown	n	3886	921	4807	
Number of Events Hospitalization after	Median (Q1–Q3)	0 (0–0)	0 (0–0)	0 (0–0)	<0.001
Mean Hospitalization Days after	Median (Q1–Q3)	4.0 (2.9–7.0)	4.0 (2.5–6.5)	4.0 (2.7–7.0)	0.4
Unknown	n	3886	921	4807	

**Table 7:** Within Treatment Groups: Healthcare Utilization and Costs.

**Within-Subject Comparison (Study)**				
**Characteristic**	**Statistic**	**Before**	**After**	***p*** **-Value**
		**N = 1226**	**N = 1226**	
Ed Visits	Median (Q1–Q3)	0 (0–1)	0 (0–0)	0.005
Health Professions	Median (Q1–Q3)	8 (4–15)	5 (2–11)	<0.001
Secondary Care	Median (Q1–Q3)	7 (3–12)	4 (1–9)	<0.001
Primary Care	Median (Q1–Q3)	20 (14–29)	14 (7–22)	<0.001
Sum Hospitalization Days	Median (Q1–Q3)	1 (1–3)	5 (3–14)	<0.001
Unknown	n	815	921	
Number of Events Hospitalization	Median (Q1–Q3)	0 (0–1)	0 (0–0)	<0.001
Mean Hospitalization Days	Median (Q1–Q3)	0.0 (0.0–4.0)	4.0 (2.5–6.5)	<0.001
Unknown	n	336	921	
**Within-Subject Comparison (Control)**				
**Characteristic**	**Statistic**	**Before**	**After**	***p*** **-Value**
		**N = 4898**	**N = 4898**	
Ed Visits	Median (Q1–Q3)	0 (0–0)	0 (0–0)	<0.001
Health Professions	Median (Q1–Q3)	5 (3–10)	3 (1–8)	<0.001
Secondary Care	Median (Q1–Q3)	6 (3–10)	4 (1–8)	<0.001
Primary Care	Median (Q1–Q3)	15 (10–21)	12 (6–18)	<0.001
Sum Hospitalization Days	Median (Q1–Q3)	1 (1–2)	6 (3–13)	<0.001
Unknown	n	3726	3886	
Number of Events Hospitalization	Median (Q1–Q3)	0 (0–0)	0 (0–0)	0.009
Mean Hospitalization Days	Median (Q1–Q3)	0.0 (0.0–3.0)	4.0 (2.9–7.0)	<0.001
Unknown	n	1812	3886	

## Data Availability

The data presented in this study are available upon request from the corresponding author due to ethical restrictions and data protection regulations.

## Abbreviations

CKD	Chronic Kidney Disease
eGFR	estimated Glomerular Filtration Rate
IQR	Interquartile Range
SD	Standard Deviation
STOPP	Screening Tool of Older Persons’ Prescriptions
START	Screening Tool to Alert to Right Treatment
